# Effectiveness of various atropine concentrations in myopia control for Asian children: a network meta-analysis

**DOI:** 10.3389/fphar.2024.1503536

**Published:** 2024-12-16

**Authors:** Xiaoyan Wang, Linyu Zhang, Jinhua Gan, Yun Wang, Weihua Yang

**Affiliations:** ^1^ School of Nursing, Southwest Medical University, Luzhou, Sichuan, China; ^2^ The Affiliated Eye Hospital, Nanjing Medical University, Nanjing, Jiangsu, China; ^3^ The Affiliated Hospital, Southwest Medical University, Luzhou, Sichuan, China; ^4^ Shenzhen Eye Institute, Shenzhen Eye Hospital, Jinan University, Shenzhen, Guangdong, China

**Keywords:** atropine, concentration, effectiveness, myopia, children, AISA

## Abstract

**Objectives:**

To assess the effectiveness of various atropine concentrations in managing myopia among children in East, South, and Southeast Asia, and to determine the most effective concentration.

**Methods:**

A systematic literature review was conducted using PubMed, Web of Science, Cochrane Library, and EMBASE. The search was limited to articles published up to 1 June 2024, and included studies in Chinese or English. Two researchers independently screened the literature, extracted relevant data, and assessed the data quality using the Revised Cochrane risk-of-bias 2 (RoB2) tool. A network meta-analysis was performed using Stata 14.2 software to compare the efficacy of different atropine concentrations in delaying myopia progression, measured by changes in refraction and axial length.

**Results:**

The analysis included 39 studies with 7,712 participants, examining 10 atropine concentrations ranging from 0.005% to 1%. Forest plots indicated that five concentrations (0.01%, 0.02%, 0.025%, 0.05%, and 1%) were more effective than a placebo in controlling myopia progression. The cumulative ordination plot indicated that 0.05% atropine most effectively delayed refraction change, which the mean change per year was 0.62D, while 1% was superior in slowing axial length progression, which the mean change per year was −0.43 mm. Considering both measures, 1% atropine showed the highest efficacy which the mean changes per year were 0.56D in spherical equivalent refraction and −0.43 mm in axial length, followed by 0.05% and 0.125% atropine.

**Conclusion:**

While 1% atropine demonstrated the highest efficacy in myopia control among East, South and Southeast Asian children, its use is not recommended due to increased adverse effects and a rapid rebound in myopia after cessation. Considering both efficacy and safety, 0.05% atropine is suggested as the optimal concentration for myopia management in this population.

## 1 Introduction

Refractive errors are a leading cause of vision impairment globally, affecting an estimated 12.8 million children aged 5-15, with the highest prevalence in Asia (Resnikoff et al., 2008). Myopia, in particular, is a predominant visual health issue among adolescents. Projections indicate that by 2050, nearly half the world’s population—4.8 billion individuals—will be myopic (Holden et al., 2016). The prevalence of myopia in East, South and Southeast Asia is nearly 90%, with a higher proportion of high myopia cases compared to the 50% prevalence rate among European youth (Morgan et al., 2018). This disparity may be due to genetic factors, as well as increased educational pressures and lifestyle changes impacting students’ eye health (Ig et al., 2012). The rising prevalence of myopia annually is linked to serious complications such as glaucoma, scleral staphyloma, retinal tears, and retinal detachment, posing significant threats to the visual health of Asian adolescents (Haarman et al., 2020). Therefore, controlling the onset of myopia in school-aged children and managing its progression is vital for reducing the risk of high myopia and its associated complications later in life (Saw et al., 2019).

Current treatments for myopia encompass spectacles, orthokeratology lenses, pharmaceutical eye drops (including atropine, pirenzepine, and timolol), and repeated low-level red-light (LLRL) therapy (Tapasztó et al., 2023). However, traditional optical interventions have shown limitations in controlling myopia progression, with diminishing effects over time (Jones et al., 2022; Lam et al., 2022; Sankaridurg et al., 2023). For example, a study by Sarkar et al. (2023) found that the maximum control effect of spectacles, soft contact lenses, and orthokeratology lenses occurred within the first 12 months of treatment. Emerging treatments like LLRL, while promising, lack robust clinical evidence due to their brief implementation period and have been associated with adverse events such as choroidal thickening, as reported in a multicenter randomized controlled trial by Jiang et al. (2022).

Atropine eye drops have emerged as a prominent method in myopia control. Clinical studies have shown that various concentrations of atropine are more effective in preventing and slowing the progression of myopia in adolescents than other single treatments (Chia et al., 2016; Yam et al., 2019; Li et al., 2021; Repka et al., 2023). A 2023 study, which analyzed results from 64 trials, concluded that high-dose topical atropine (≥0.5%) is the most effective intervention for reducing the axial elongation rate (Lawrenson et al., 2023). Research by Li et al. (2014) further supports the significant slowing effect of atropine on the progression of myopia in children, particularly in Asian children. As atropine’s use expands in myopia prevention and control among adolescents across different Asian economies, the selection of individualized concentrations and consideration of potential side effects are crucial for clinical decisions. While previous meta-analyses have evaluated the efficacy of different atropine concentrations (Gong et al., 2017; Fu et al., 2020; Wang et al., 2024), a systematic review or meta-analysis comprehensively summarizing its effects on the East, South and Southeast Asian population has been lacking. Thus, leveraging the latest randomized controlled trials (RCTs), we conducted a network meta-analysis to assess the impact of different concentrations of atropine eye drops on key ocular anatomical parameters such as spherical equivalent refraction (SER) and axial length (AL) in Asian children.

## 2 Materials and methods

This study strictly adhered to the guidelines of the Preferred Reporting Items for Systematic Reviews incorporating Network Meta-Analyses (PRISMA-NMA) and was registered with the International Prospective Register of Systematic Reviews (PROSPERO) under the identifier CRD42024551014 (Hutton et al., 2015).

### 2.1 Inclusion and exclusion criteria

The study included randomized clinical trials (RCTs) and observational studies that met the following criteria:

(ⅰ). Participants were East, South and Southeast Asian individuals under 18 years old with myopia (spherical equivalent ≤ −0.5 diopters).
(ⅱ). Studies utilized at least one concentration of atropine eye drops, with controls including other treatments for myopia, placebos, monovision lenses, or no treatment.
(ⅲ). They reported outcomes related to myopia progression (e.g., changes in refraction and axial length) and/or side effects of atropine therapy.
(ⅳ). Ethical review board approval was documented.

Exclusions applied to studies not available in full text, non-original articles like case reports, reviews, conference abstracts, editorials, letters, non-human studies, and those with duplicate or non-applicable data.

### 2.2 Search strategy

A systematic search was conducted across four databases—PubMed, Web of Science, Cochrane Library, and EMBASE—focusing on RCTs and observational studies concerning atropine concentrations for myopia control in children. The search spanned from database inception to 1 June 2024, and was limited to Chinese or English language publications. Search terms encompassed “atropine,” “atropine sulfate,” “cholinergic antagonists,” “atropinol,” “myopia,” “nearsightedness,” and “shortsightedness.” Adjustments were made for database-specific requirements, and relevant review and meta-analysis literature was traced for additional literature. Detailed search strategies are provided in the Supplementary Tables S1–S4.

### 2.3 Study selection and data extraction

Zotero software facilitated the management of retrieved literature. Two researchers independently screened articles based on the inclusion and exclusion criteria, initially reviewing titles and abstracts, followed by full-text assessments. Any discrepancies were resolved through discussion or by a third party, who also evaluated the risk of bias and study quality. Extracted data encompassed first author, publication year, study location, participant demographics, follow-up duration, intervention details, sample size, baseline characteristics, mean change data, and adverse reactions.

### 2.4 Risk of bias assessment

The Revised Cochrane risk-of-bias 2 (RoB2) tool was used to assess five bias aspects: the randomization process, deviations from intended interventions, missing outcome data, outcome measurement, and selection of reported results. Each aspect was rated as ‘high risk’, ‘low risk’, or ‘some concerns' (Jp et al., 2011). Two researchers independently evaluated the studies, with final consensus reached through discussion.

### 2.5 Statistical analyses

Refraction and axial length, being continuous variables, were analyzed using mean difference (MD) as the effect size, with each study’s effect size presented as a 95% confidence interval (CI). The significance level was set at 0.05. Network Meta-analysis was conducted using the network package of Stata 14.2 software. We used heterogeneity variance parameter (τ^2^) and generalized Cochran’s Q to assess heterogeneity between studies. If there is heterogeneity, we use a random-effects model based on a frequentist approach, and conversely, we will use a fixed-effects model. Inconsistency was assessed using the inconsistency model and node-splitting method. Node splitting is an effective method used to test for local inconsistency in network meta-analysis. The method determined the presence of local inconsistencies by splitting nodes (i.e., different atropine concentrations) in a network into multiple parts, thereby assessing the impact of these different parts on the results separately. Compared with the inconsistency model, it can reveal local sources of inconsistency in a finer way, which helped to understand and explain the discrepancies in the results of the study, and ensured the reliability and stability of the results of the network meta-analysis. A *p*-value > 0.05 indicated no significant inconsistency. If inconsistency was minimal, the consistency model was applied. Loop inconsistency was also tested; a small inconsistency factor (IF) with a 95% CI including 0 indicated no significant loop inconsistency, ensuring stability and reliability of direct and indirect comparisons. Network plots were constructed, with line thickness indicating the number of direct comparisons and node size reflecting sample size. The Surface Under the Cumulative Ranking (SUCRA) was calculated to rank treatments, with values closer to one indicating higher efficacy. Funnel plots and egger’s test were used to assess publication bias, with asymmetry showing potential bias. Forest plots and league tables were also generated.

## 3 Results

### 3.1 Basic literature characteristics

**FIGURE 1:**
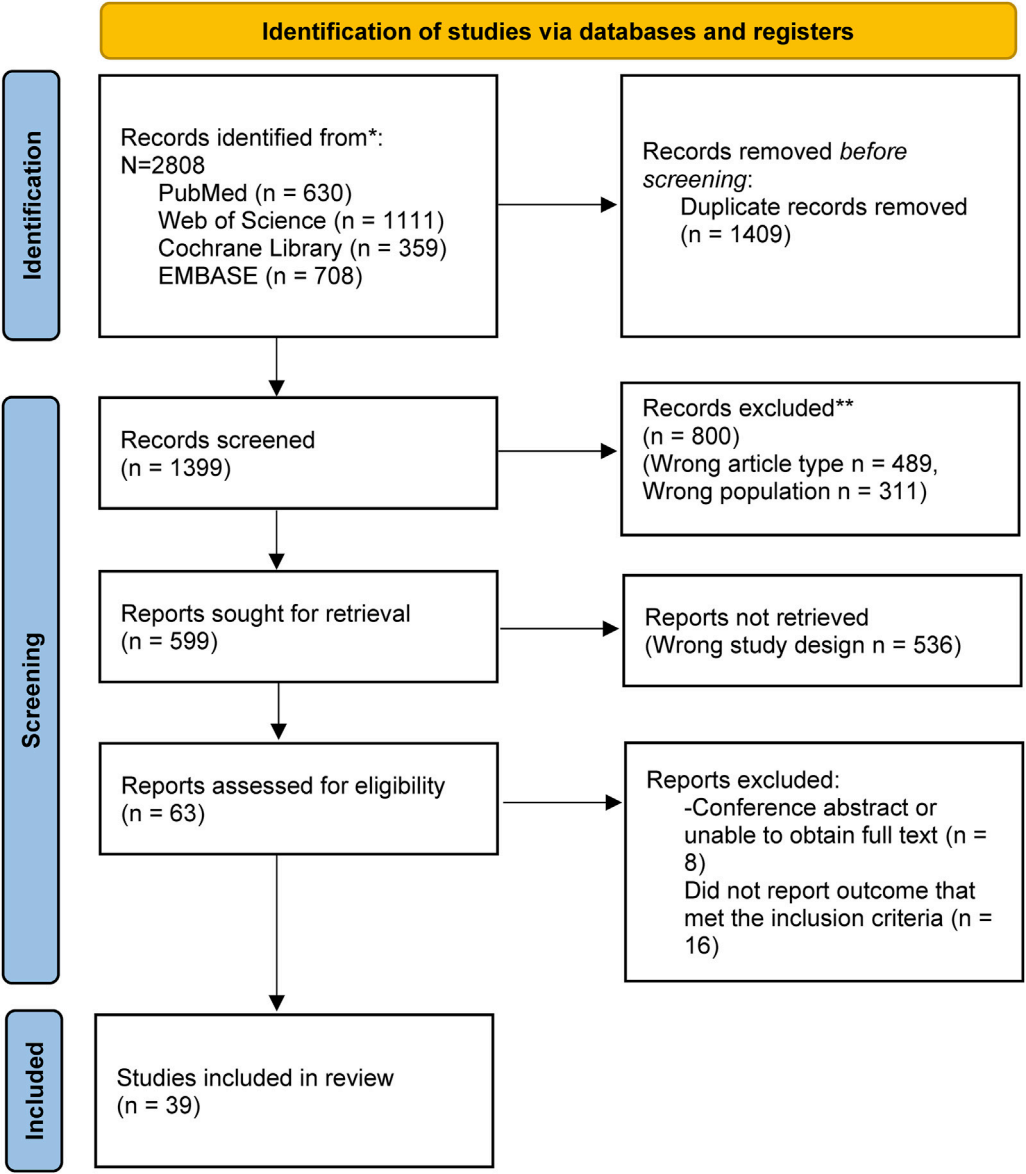
PRISMA flow diagram shows the study process. PRISMA: Preferred reporting items for systematic review and Meta-analysis.

Our systematic search across PubMed, Web of Science, Cochrane Library, and EMBASE yielded 2,808 documents related to the study topic. After excluding 1,409 duplicates, a full-text evaluation of the remaining 1,399 documents led to the exclusion of 1,360 studies due to incompatibility with the study criteria or other reasons. This rigorous process resulted in the inclusion of 39 eligible studies, the details of which are depicted in the PRISMA flowchart (Figure 1).

These 39 studies encompassed a total of 7,712 participants, with 2,146 in the non-atropine treatment group and 5,566 in the atropine treatment group. The studies spanned a publication period from 2006 to 2024 and were conducted among East, South and Southeast Asian populations. The follow-up duration varied from 3 to 54 months, encompassing children aged 4–15 years with varying severities of myopia. Treatments involved 10 different atropine concentrations: 0.005%, 0.0025%, 0.01%, 0.02%, 0.025%, 0.05%, 0.1%, 0.125%, 0.5%, and 1%. Most studies provided data on baseline refraction and axial length (AL), as well as mean changes in these parameters. However, only one study reported adverse effects, and there were gaps in the data: two studies lacked mean change in refraction, and three studies did not report both mean change in refraction and AL. A comprehensive list of the included data are presented in Table 1.

**TABLE 1:** General information of included studies.

Study	Region	Age (y)	Follow-up	Arm	Sample size	Baseline refraction (D)	Baseline AL (mm)	Mean change in refraction (D/y)	Mean change in AL (mm/y)	Side effects
Zhu et al. (2023c)	China	7–12	2 years	0.05% atropine	72	−3.26 ± 0.20	23.71 ± 0.23	−0.46 ± 0.30	0.26 ± 0.30	NA
				Placebo	70	−3.27 ± 0.32	23.69 ± 0.19	−1.72 ± 1.12	0.76 ± 0.62	NA
Aicun et al. (2022)	China	6–12	1 year	0.01% atropine	119	−2.70 ± 1.64	24.58 ± 0.74	−0.47 ± 0.32	0.37 ± 0.20	Photophobia, glare and blurred vision
				0.02% atropine	117	−2.76 ± 1.47	24.60 ± 0.72	−0.38 ± 0.35	0.30 ± 0.17	Photophobia, glare and blurred vision
Kumaran et al. (2015)	Singapore	6–12	3 years	1% atropine	147	−3.36	24.80	−0.45 ± 0.84	0.0867 ± 0.27	NA
				Placebo	166	−3.58	24.80	−0.517 ± 0.8	0.1767 ± 0.46	NA
Wang et al. (2020)	China	6–14	6 m	0.01% atropine	38	−1.94 ± 1.17	24.21 ± 0.9	−0.6 ± 0.42	0.48 ± 0.16	NA
				Placebo	25	−1.78 ± 1.15	24.33 ± 0.64	−1.2 ± 0.43	0.7 ± 0.20	NA
Chia et al. (2014)	Singapore	6–12	3 years	0.01% atropine	75	−4.47 ± 1.5	25.17 ± 0.98	−0.28 ± 0.72	0.19 ± 0.38	NA
				0.1% atropine	141	−4.49 ± 1.45	25.13 ± 0.83	−0.35 ± 0.83	0.30 ± 0.38	NA
				0.5% atropine	140	−4.33 ± 1.83	25.14 ± 0.92	−0.41 ± 0.81	0.21 ± 0.35	NA
Chia et al. (2012)	Taiwan	6–12	2 years	0.01% atropine	75	−4.5 ± 1.5	25.2 ± 1.0	−0.49 ± 0.63	0.41 ± 0.32	NA
				0.1% atropine	141	−4.5 ± 1.4	25.1 ± 0.8	−0.38 ± 0.6	0.28 ± 0.27	NA
				0.5% atropine	139	−4.3 ± 1.8	25.1 ± 0.9	−0.30 ± 0.6	0.27 ± 0.25	NA
Chua et al. (2006)	Singapore	6–12	2 years	1% atropine	166	−3.58 ± 1.17	24.80 ± 0.84	−0.28 ± 0.92	−0.02 ± 0.35	NA
				Placebo	190	−3.36 ± 1.38	24.80 ± 0.83	−1.20 ± 0.69	0.38 ± 0.38	NA
Chia et al. (2023)	Singapore	6–11	1 year	0.0025% atropine	22	−3.00 ± 1.1	24.35 ± 0.8	−0.55 ± 0.337	0.3	NA
				0.005% atropine	24	−3.8 ± 1.4	24.64 ± 0.8	−0.33 ± 0.473	0.27 ± 0.15	NA
				0.01% atropine	25	−3.25 ± 1.1	24.77 ± 0.7	−0.39 ± 0.519	0.25 ± 0.25	NA
				Placebo	26	−3.93 ± 1.31	24.79 ± 0.8	−0.55 ± 0.471	0.35 ± 0.17	NA
Zhao and Hao (2021)	China	5–14	1 year	0.01% atropine	20	−1.98 ± 0.45	24.17 ± 0.68	−0.34 ± 0.16	0.24 ± 0.12	NA
				spectacles	20	−1.93 ± 0.74	24.28 ± 0.83	−1.30 ± 0.44	0.72 ± 0.21	NA
Rong et al. (2020)	China	6–14	1 year	0.01% atropine	62	−2.39 ± 1.54	24.43 ± 0.87	−0.46 ± 0.42	0.36 ± 0.21	NA
				spectacles	68	−2.15 ± 1.47	24.34 ± 0.70	−0.7 ± 0.42	0.46 ± 0.41	NA
Qin et al. (2021)	China	6–14	1 year	0.02% atropine	92	−2.57 ± 1.37	24.32 ± 0.77	−0.46 ± 0.49	0.38 ± 0.21	NA
				0.01% atropine	101	−2.68 ± 1.59	24.51 ± 0.76	−0.48 ± 0.46	0.39 ± 0.19	NA
Han et al. (2019)	China	6–12	2 y	1% atropine	53	−1.74 ± 1.4	24.30 ± 0.99	NA	0.32 ± 1.00	NA
				no medication	25	−1.81 ± 1.01	24.04 ± 0.65	NA	1.52 ± 0.68	NA
Li et al. (2020)	Hong Kong	4–12	1 year	0.05% atropine	102	−3.95 ± 1.64	24.86 ± 0.9	−0.27 ± 0.61	0.20 ± 0.25	NA
				0.025% atropine	91	−3.83 ± 1.81	24.92 ± 0.89	−0.46 ± 0.45	0.29 ± 0.2	NA
				0.01% atropine	97	−3.95 ± 1.9	24.79 ± 1.02	−0.59 ± 0.61	0.36 ± 0.29	NA
				Placebo	93	−4.1 ± 1.9	24.9 ± 0.99	−0.81 ± 0.53	0.41 ± 0.22	NA
Wang et al. (2023a)	China	6–14	2 years	0.02% atropine	105	−2.81 ± 1.47	24.61 ± 0.69	−0.06 ± 0.40	0.05 ± 0.21	NA
				0.01% atropine	106	−2.76 ± 1.56	24.60 ± 0.72	−0.09 ± 0.41	0.08 ± 0.53	NA
				SV spectacles	89	−2.66 ± 1.39	24.54 ± 0.69	−0.12 ± 0.28	0.13 ± 0.72	NA
Liang et al. (2023)	China	6–12	1 year	0.01% atropine	38	−2.69 ± 1.27	24.64 ± 0.78	−0.45 ± 0.44	0.30 ± 0.19	NA
				Placebo	41	−2.85 ± 1.45	24.54 ± 0.99	−0.75 ± 0.50	0.43 ± 0.20	NA
				0.01% atropine	38	−2.29 ± 1.22	24.47 ± 0.80	−0.56 ± 0.44	0.30 ± 0.19	NA
				Placebo	42	−2.41 ± 1.53	24.54 ± 0.99	−0.72 ± 0.51	0.39 ± 0.21	NA
Sen et al. (2022)	India	5–15	2 years	0.01% atropine	72	−3.92 ± 1.009	24.54 ± 0.64	−0.145 ± 0.31	0.0575 ± 0.11	NA
				Placebo	73	−4.05 ± 1.25	24.58 ± 0.79	−0.438 ± 0.217	0.1515 ± 0.12	NA
Sen et al. (2022)	India	5–15	2 y	0.01% atropine	72	−3.76 ± 1.54	24.5 ± 0.66	0.18 ± 0.308	0.0575 ± 0.127	NA
				Placebo	73	−4.13 ± 1.237	24.49 ± 0.65	0.4485 ± 0.238	0.153 ± 0.13	NA
Yam et al. (2023)	Hong Kong	4–9	2 years	0.05% Atropine	116	0.50 ± 0.33	22.82 ± 0.72	−0.23 ± 0.69	0.24 ± 0.30	NA
				0.01% atropine	122	0.51 ± 0.33	22.89 ± 0.70	−0.42 ± 0.79	0.32 ± 0.36	NA
				Placebo	115	0.53 ± 0.31	22.80 ± 0.64	−0.51 ± 0.77	0.35 ± 0.33	NA
Fu et al. (2020)	China	6–14	1 year	0.02% atropine	117	−2.76 ± 1.47	24.60 ± 0.72	−0.11 ± 0.33	0.30 ± 0.28	NA
				0.01% atropine	119	−2.70 ± 1.64	24.58 ± 0.74	−0.15 ± 0.33	0.35 ± 0.22	NA
				SV spectacles	100	−2.68 ± 1.42	24.55 ± 0.71	−0.23 ± 0.28	0.49 ± 0.31	NA
Cui et al. (2023)	China	6–14	1 year	0.01% atropine	119	−2.70 ± 1.64	24.58 ± 0.74	−0.47 ± 0.45	0.37 ± 0.22	NA
				SV spectacles	100	−2.68 ± 1.42	24.55 ± 0.71	−0.7 ± 0.6	0.46 ± 0.35	NA
Zhu et al. (2023b)	China	6–14	9 m	0.01% atropine	69	−2.18 ± 1.19	24.57 ± 0.95	−0.467 ± 0.33	0.267 ± 0.19	NA
				single-vision lenses	50	−2.05 ± 1.48	24.42 ± 0.81	−0.7467 ± 0.49	0.44 ± 0.19	NA
Hieda et al. (2021)	Japan	6–12	2 years	0.01% atropine	77	−2.92 ± 1.43	24.41 ± 0.86	−0.63 ± 0.40	0.315 ± 0.18	NA
				Placebo	81	−2.96 ± 1.24	24.50 ± 0.69	−0.74 ± 0.41	0.385 ± 0.18	NA
Zhu et al. (2020)	China	6–12	4 years	1% atropine	262	−3.82 ± 0.44	24.93 ± 0.21	−0.41 ± 0.23	0.19 ± 0.13	NA
				Placebo	308	−3.74 ± 0.51	24.91 ± 0.18	−0.75 ± 0.64	0.40 ± 0.16	NA
Ye et al. (2022)	China	6–12	1 year	1% atropine	91	−2.10 ± 1.1	24.32 ± 0.83	−0.58 ± 0.36	0.06 ± 0.12	NA
				0.01% atropine	80	−2.20 ± 1.13	24.26 ± 0.74	−0.56 ± 0.34	0.36 ± 0.12	NA
Chan et al. (2022)	Hong Kong	7–10	1.5 y	0.01% atropine	34	−1.88 ± 1.08	24.17 ± 0.79	−0.7 ± 0.39	0.32 ± 0.16	NA
				Placebo	27	−1.74 ± 0.71	24.09 ± 0.74	−0.66 ± 0.41	0.30 ± 0.22	NA
Chaurasia et al. (2022)	India	6–16	1 year	0.01% atropine	43	−3.04 ± 1.36	24.52 ± 1.94	−0.26 ± 0.23	0.2 ± 0.21	NA
				0.5% carboxymethyl cellulose	43	−3.07 ± 1.32	24.56 ± 1.84	−0.72 ± 0.29	0.36 ± 0.24	NA
Kao et al. (2021)	Taiwan	6–15	1 year	0.125% atropine	20	−1.25 ± 0.94	23.91 ± 1.02	−0.14 ± 1.07	0.06 ± 1.11	NA
				Untreated	20	0.15 ± 1.29	23.35 ± 0.92	−0.82 ± 1.29	0.35 ± 0.97	NA
Zhang X.J. et al. (2024)	Hong Kong	4–12	2 years	0.05% atropine	68	−3.95 ± 1.64	43.77 ± 1.45	−0.27 ± 0.88	0.20 ± 0.88	NA
				0.025% atropine	59	−3.83 ± 1.81	43.71 ± 1.28	−0.46 ± 0.71	0.27 ± 0.32	NA
				0.01% atropine	79	−4.1 ± 1.9	43.91 ± 1.32	−0.585 ± 0.93	0.32 ± 0.39	NA
Fang et al. (2010)	Taiwan	6–12	18.4 m	0.025% Atropine	24	−0.31 ± 0.45	NA	−0.14 ± 0.24	NA	NA
			16.3 m	untreated	26	−0.17 ± 0.50	NA	−0.58 ± 0.34	NA	NA
Lee et al. (2006)	Taiwan	6–12	19.9 m	0.05% atropine	21	−1.58 ± 1.37	NA	−0.28 ± 0.26	NA	NA
				untreated	36	−1.41 ± 0.86	NA	−0.75 ± 0.35	NA	NA
Wang et al. (2023b)	China	6–12	6 m	0.01% atropine	30	−0.19 ± 0.28	23.59 ± 0.77	−0.30 ± 0.26	0.34 ± 0.11	NA
				Placebo	30	−0.21 ± 0.32	23.61 ± 0.75	−0.68 ± 0.34	0.56 ± 0.14	NA
Fu et al. (2021)	China	6–14	1 year	0.02% atropine	138	−2.79 ± 1.43	24.61 ± 0.72	NA	0.3 ± 0.22	NA
				0.01% atropine	141	−2.76 ± 1.61	24.59 ± 0.74	NA	0.36 ± 0.19	NA
Cui et al. (2021)	China	6–14	2 years	0.02% atropine	105	−2.81 ± 1.47	24.61 ± 0.69	−0.40 ± 0.52	0.31 ± 0.29	NA
				0.01% atropine	106	−2.76 ± 1.56	24.60 ± 0.72	−0.465 ± 0.59	0.36 ± 0.31	NA
				Placebo	89	−2.66 ± 1.39	24.54 ± 0.69	−0.66 ± 0.72	0.44 ± 0.35	NA
Pan et al. (2022)	China	6–12	6 m	0.01% atropine	125	−1.64 ± 0.8	24.13 ± 0.76	−0.54 ± 0.33	0.38 ± 0.14	NA
				untreated	60	−1.59 ± 0.94	24.06 ± 0.77	−1.20 ± 0.35	0.52 ± 0.14	NA
Wang et al. (2022)	China	6–14	3 m	0.01% atropine	21	−2.38 ± 1.46	24.45 ± 1.06	−0.44 ± 1.04	0.16 ± 0.48	NA
				untreated	19	−2.36 ± 1.87	24.70 ± 0.93	−1.04 ± 0.92	0.48 ± 0.36	NA
Moon and Shin (2018)	South Korea	5–14	13.7 m	0.01% atropine	89	−3.84 ± 2.47	24.86 ± 1.22	−0.84 ± 0.86	0.444 ± 0.32	NA
			10.5 m	0.025% atropine	63	−3.97 ± 1.65	24.66 ± 0.93	−0.564 ± 0.86	0.3 ± 0.24	NA
			14.0 m	0.05% atropine	133	−3.94 ± 2.76	24.91 ± 1.43	−0.228 ± 0.67	0.228 ± 0.25	NA
Wu et al. (2011)	Taiwan	6–12	4.54 y	0.05% atropine	97	−2.45 ± 1.63	NA	−0.31 ± 0.26	NA	NA
			4.11 y	untreated	20	−1.87 ± 0.94	NA	−0.90 ± 0.30	NA	NA
Yi et al. (2015)	China	7–12	1 year	1% atropine	68	−1.23 ± 0.32	23.75 ± 0.12	0.32 ± 0.22	−0.03 ± 0.07	NA
				placebo eyedrops	64	−1.15 ± 0.3	23.72 ± 0.12	−0.85 ± 0.31	0.32 ± 0.15	NA
Yam et al. (2022)	Hong Kong	4–12	1 year	0.05% atropine	45	−4.49 ± 1.95	25.13 ± 0.9	−0.28 ± 0.42	0.17 ± 0.14	NA
				0.025% atropine	39	−5.11 ± 2.47	25.53 ± 1.0	−0.35 ± 0.37	0.20 ± 0.15	NA
				0.01% atropine	43	−5.65 ± 3.04	25.49 ± 1.33	−0.38 ± 0.49	0.24 ± 0.18	NA
Yam et al. (2020)	Hong Kong	4–12	2 years	0.05% atropine	93	−3.93 ± 1.63	24.88 ± 0.91	−0.275 ± 0.86	0.195 ± 0.35	NA
				0.025% atropine	86	−3.88 ± 1.83	24.94 ± 0.9	−0.425 ± 0.73	0.25 ± 0.33	NA
				0.01% atropine	91	−3.99 ± 1.94	24.78 ± 1.02	−0.56 ± 0.77	0.295 ± 0.38	NA

### 3.2 Risk of bias

**FIGURE 2:**
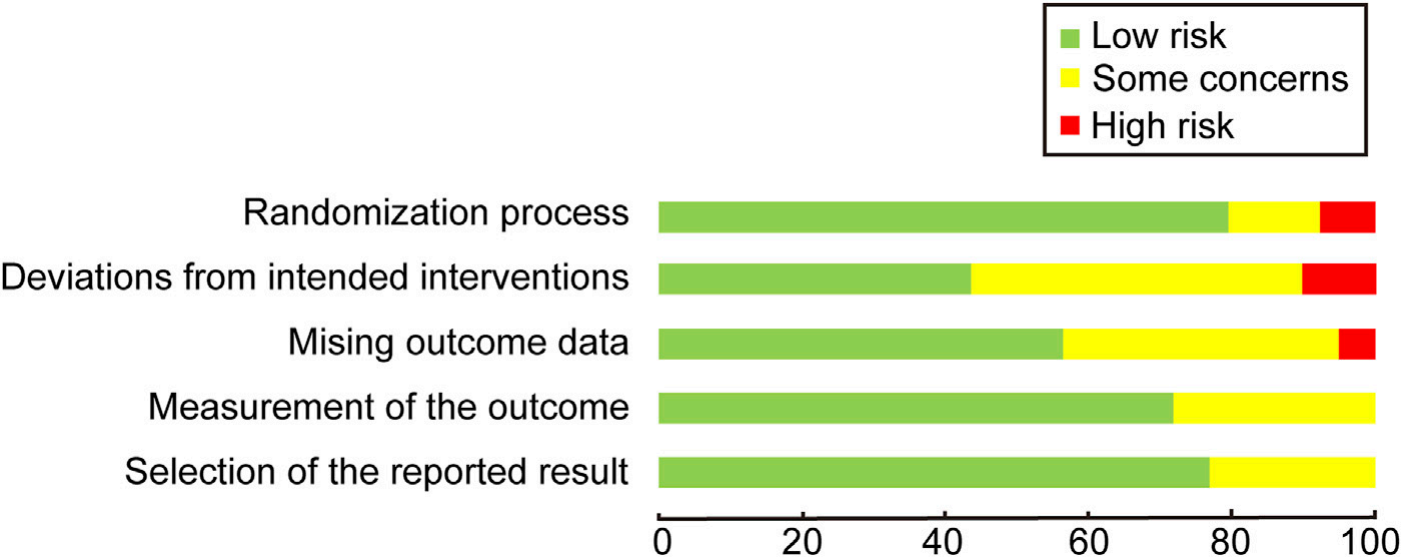
Quality assessment of included trials: risk of bias maps.

**FIGURE 3:**
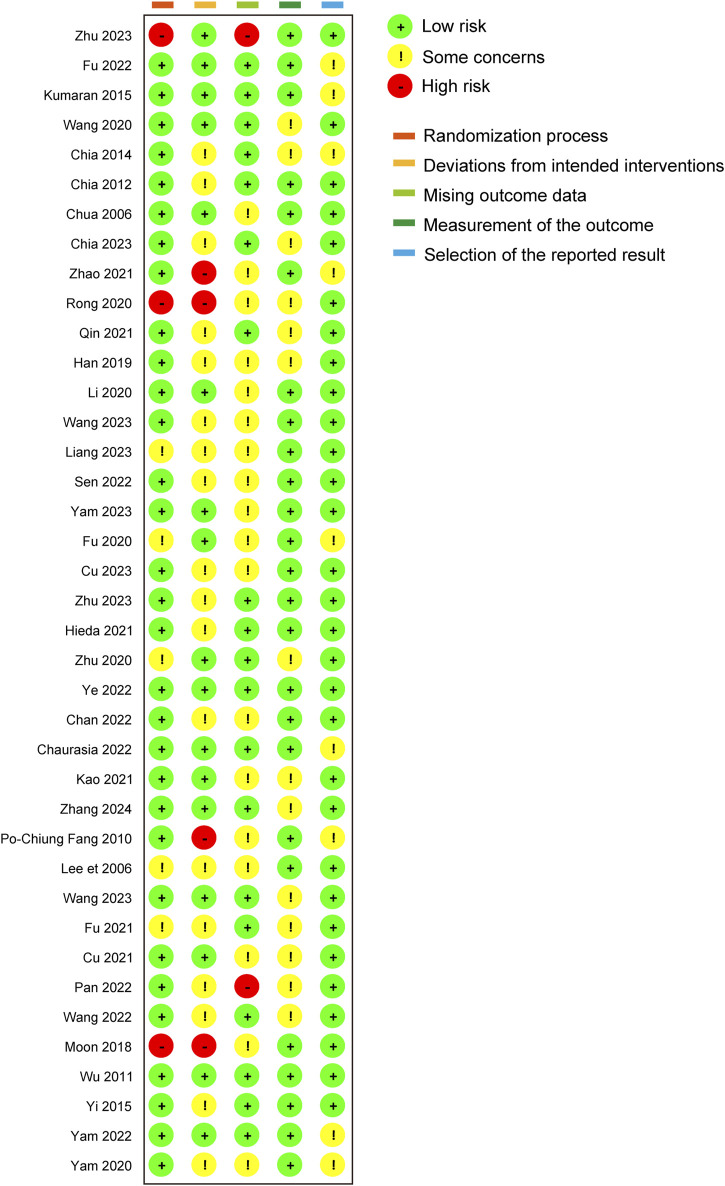
Quality assessment of included trials: summary of risk of bias.

In the comprehensive assessment of the included studies, 6 out of the 39 studies were identified to have a high risk of bias, primarily due to issues in the randomization process and deviations from the intended interventions. The most common source of bias observed was the deviation from the intended interventions during subject allocation. This may produce selection bias and performance bias. Selection bias may lead to an inaccurate response to the efficacy of atropine, affecting the representativeness of the sample, and performance bias may result in erroneous data being collected, affecting the veracity and reliability of the study results. However, outcome measurement and result selection were relatively standardized across the studies. Only 2 studies managed to achieve a low risk of bias, indicating that the majority of the trials exhibited varying degrees of concern regarding bias. The visual representation of these risk assessments is provided in Figures 2, 3.

### 3.3 Results of statistical analyses

We excluded missing data directly from the analysis when we conducted our analyses.

#### 3.3.1 Overview of network

**FIGURE 4:**
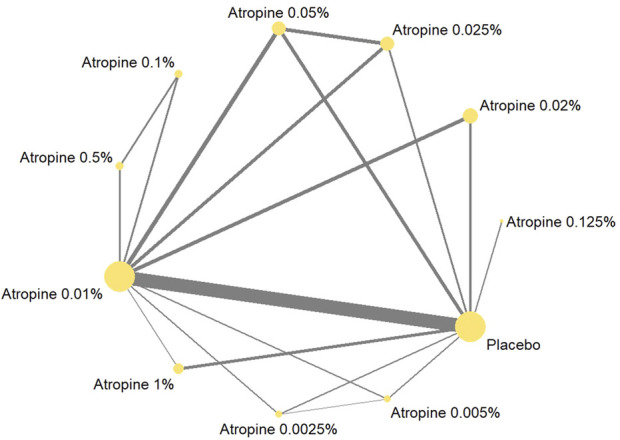
Network plot about refraction change.

In our network analysis, 37 studies reported data on changes in refraction following the intervention. Among these, 27 were two-armed studies, and 10 were multi-armed studies. The studies evaluated 10 different concentrations of atropine concentrations: 0.05%, 0.01%, 0.02%, 1%, 0.5%, 0.1%, 0.005%, 0.0025%, 0.025%, and 0.125%. The network plot for refraction change revealed that the 0.01% atropine concentration had a larger node area compared to placebo, indicating a larger total sample size for this concentration. Additionally, the thicker connecting line between 0.01% atropine and placebo in the network plot revealed a greater number of direct comparisons between these two treatments, as illustrated in Figure 4.

**FIGURE 5:**
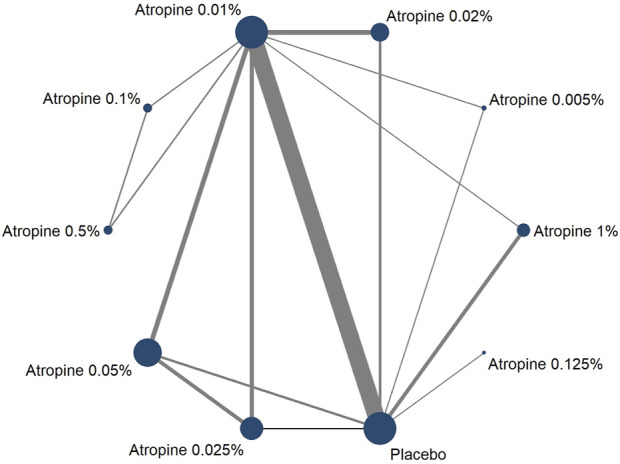
Network plot about axial length change.

Thirty-six studies reported changes in axial length following the intervention, with 27 being two-armed and 9 being multi-armed studies. Nine different atropine concentrations were considered in these analyses, but the 0.0025% concentration was not included in the axial length studies. The network plot for the change showed larger node areas for the 0.01% atropine concentration, placebo, 0.05% atropine concentration, and 0.025% atropine concentration, indicating a larger total sample size for these concentrations. The thicker connecting line between 0.01% atropine and placebo in this plot also indicated a greater number of direct comparisons, mirroring the pattern observed in the network plot, as shown in Figure 5.

#### 3.3.2 Heterogeneity and inconsistency analysis

The results of heterogeneity showed τ^2^ of 0.0579 and Cochran’s Q of 534.84 (*p* < 0.05) for change in refraction and τ^2^ of 0.0121 and Cochran’s Q of 475.76 (*p* < 0.05) for change in axial length, suggesting that there was heterogeneity among the studies. Therefore, we will choose a random effects model based on the frequentist approach for our analysis.

The global inconsistency of the network meta-analysis was assessed using Stata 14.2’s inconsistency model. The resulting *p*-value of 0.7882, which is greater than 0.05, indicated that the global inconsistency is not significant. Further analysis of local inconsistency using the node splitting method also revealed *p*-values greater than 0.05 for each study, reporting that local inconsistencies are negligible. This implied that the discrepancies between direct and indirect comparison results were minimal. Consequently, the consistency model was appropriate for this study’s analysis. Additionally, the loop inconsistency test results showed small inconsistency factors (IFs) close to zero for each closed loop, with confidence intervals that include zero. This indicated that loop inconsistency was not significant, further supporting the stability and reliability of both direct and indirect comparison outcomes, as detailed in the Supplementary Figure S1 and Figure 2.

#### 3.3.3 Indirect and direct comparisons

**FIGURE 6:**
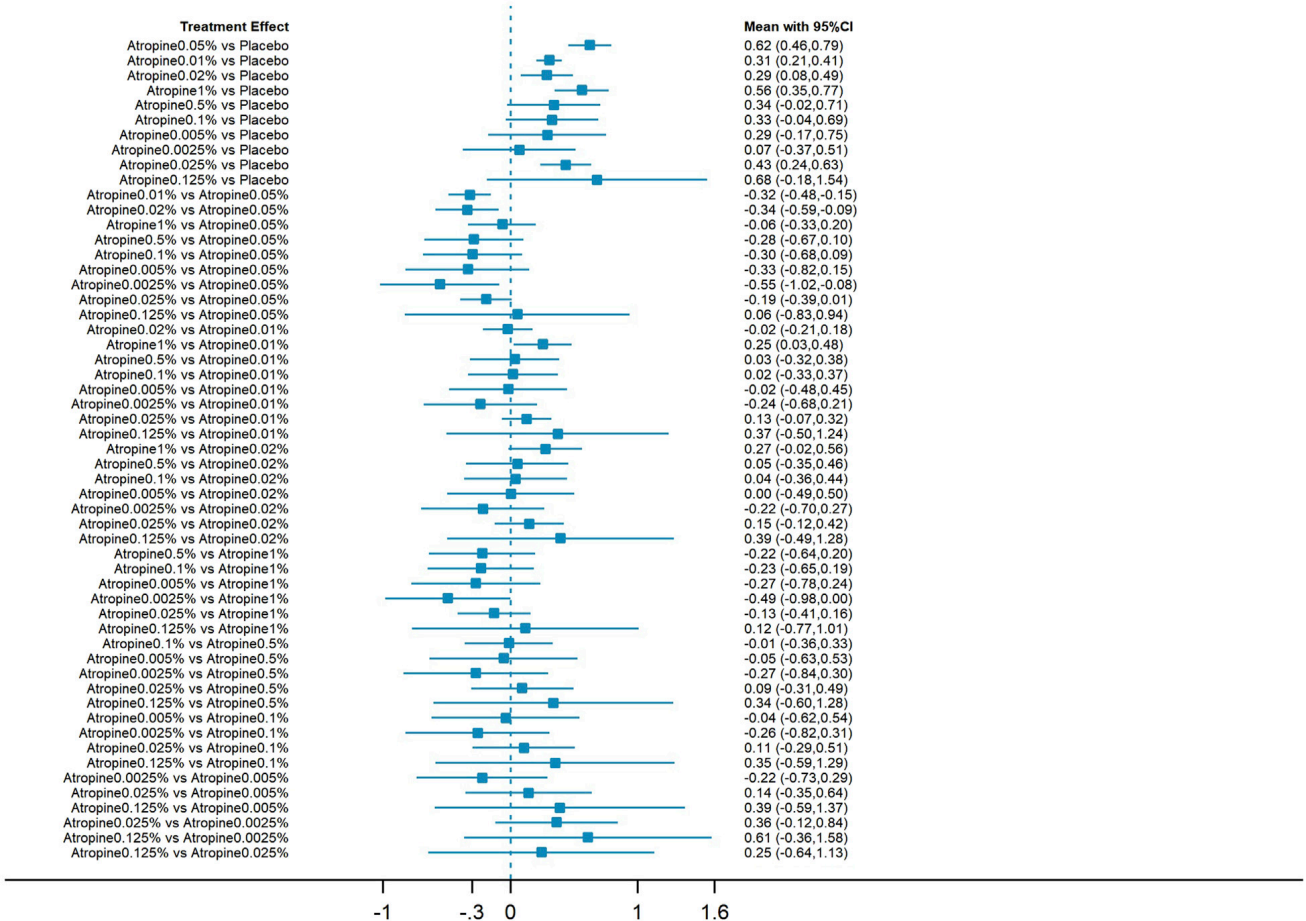
Forest plot contrasting various atropine doses for refraction change. CI: Confidence interval.

The forest plot, which included 55 pairs of two-by-two comparisons with refraction change as the outcome metric, was depicted in Figures 6, 8. In comparisons against placebo, the mean changes per year and their 95% confidence intervals (CIs) for atropine concentrations at 0.025%, 1%, 0.02%, 0.01%, and 0.05% were 0.43D/year (0.24, 0.63), 0.56D/year (0.35, 0.77), 0.29D/year (0.08, 0.49), 0.31D/year (0.21, 0.41), and 0.62D/year (0.46, 0.79), respectively. All *p*-values were less than 0.05, indicating that these atropine concentrations were more effective in delaying refraction progression than placebo. When compared to 0.05% atropine, the mean changes per year and their 95% confidence intervals for 0.01%, 0.02%, and 0.0025% atropine were −0.32D/year (−0.48, −0.15), −0.34D/year (−0.59, −0.09), and −0.55D/year (−1.02, −0.08), respectively, with *p*-values less than 0.05, reflecting these concentrations were less effective than 0.05% atropine. The comparison of 1% atropine against 0.01% showed a mean change of 0.25D/year (0.03, 0.48) with a *p*-value less than 0.05, indicating that 1% atropine was more effective in delaying refraction progression.

**FIGURE 7:**
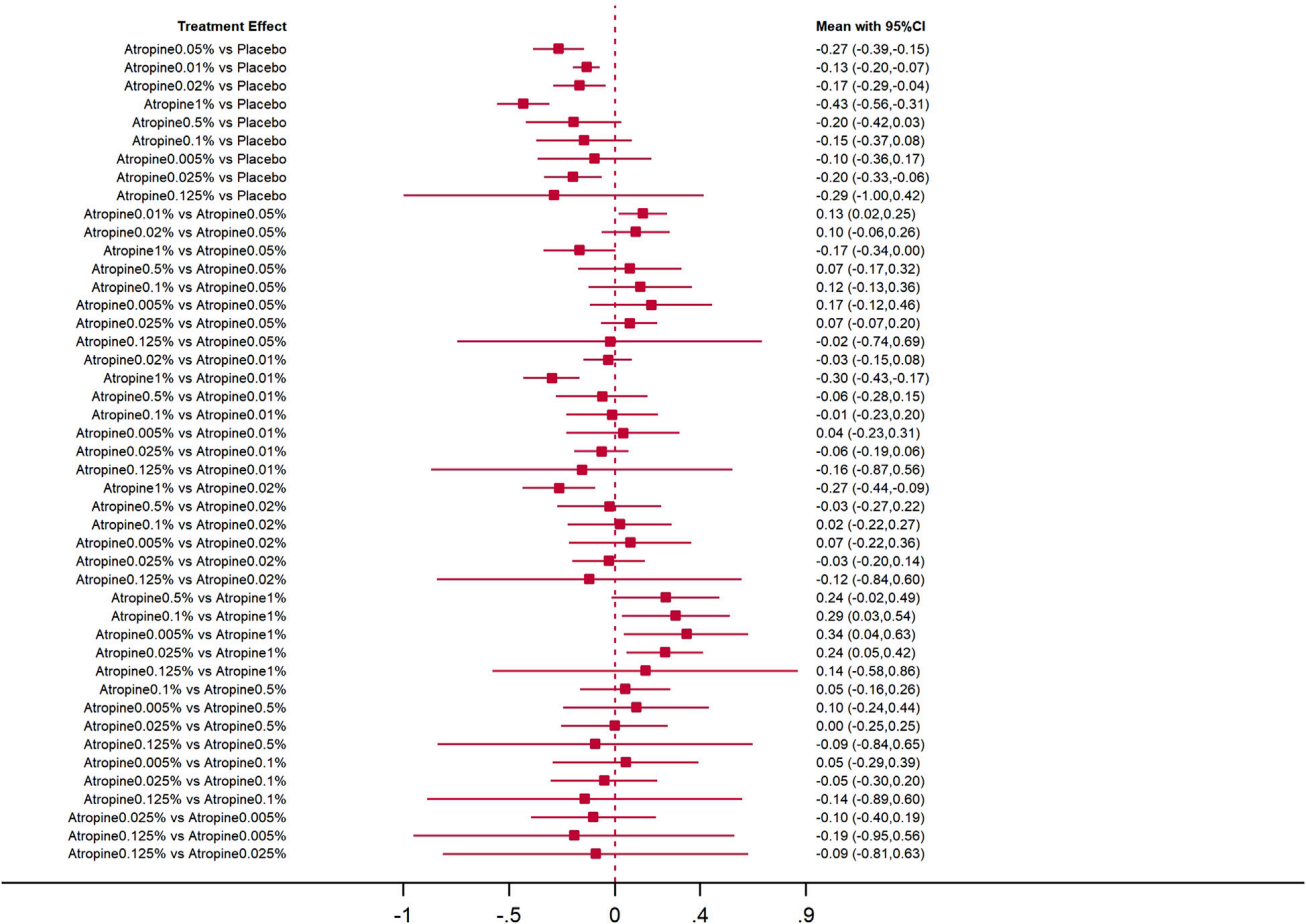
Forest plot contrasting various atropine doses for axial length change. CI: Confidence interval.

**FIGURE 8:**
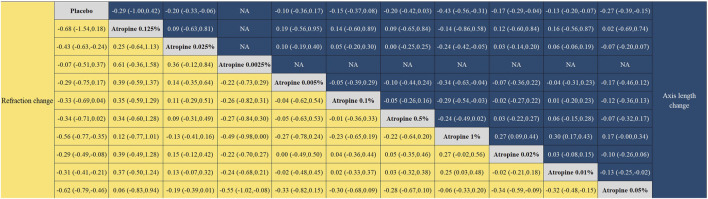
League plot for refraction change and axial length change.

The forest plot with 45 pairs of two-by-two comparisons using axial length change as the outcome metric is shown in Figures 7, 8. In placebo comparisons, the mean changes per year and their 95% confidence intervals for atropine concentrations at 0.05%, 0.01%, 0.02%, 1%, and 0.025% were −0.27 mm/year (−0.39, −0.15), −0.13 mm/year (−0.20, −0.07), −0.17 mm/year (−0.29, −0.04), −0.43 mm/year (−0.56, −0.31), and −0.20 mm/year (−0.33, −0.06), respectively, with *p*-values less than 0.05, indicating these atropine concentrations were more effective in slowing axial length progression than placebo. The comparison of 0.01% atropine against 0.05% showed a mean change of 0.13 mm/year (0.02, 0.25) with a *p*-value less than 0.05, suggesting 0.01% atropine was less effective. The comparison of 1% atropine against 0.01% showed a mean change of −0.30 mm/year (−0.43, −0.17) with a *p*-value less than 0.05, indicating 1% atropine was more effective. When comparing 1% atropine with 0.02%, the mean change was −0.27 mm/year (−0.44, −0.09) with a *p*-value less than 0.05, showing 1% atropine was more effective. In comparisons with 1% atropine, the means and 95% confidence intervals for 0.1%, 0.005%, and 0.025% atropine were 0.29 mm (0.03, 0.54), 0.34 mm (0.04, 0.63), and 0.24 mm/year (0.05, 0.42), respectively, with *p*-values less than 0.05, indicating these concentrations were less effective than 1% atropine in slowing axial length progression.

#### 3.3.4 Rank probability

The Surface Under the Cumulative Ranking (SUCRA) analysis provided insights into the relative efficacy of different atropine concentrations and placebo in managing myopia. For changes in refraction, 0.05% atropine was the most effective, with a SUCRA value of 88.7%, ranking it first among all treatments. The efficacy ranking, from highest to lowest, was as follows: 0.05% atropine, 1% atropine (80.3%), 0.125% atropine (75.5%), 0.025% atropine (63.1%), 0.5% atropine (48.4%), 0.1% atropine (46.7%), 0.005% atropine (42.8%), 0.01% atropine (41.6%), 0.02% atropine (39%), 0.0025% atropine (17.7%), and placebo with the lowest efficacy at 6.4%.

**FIGURE 9:**
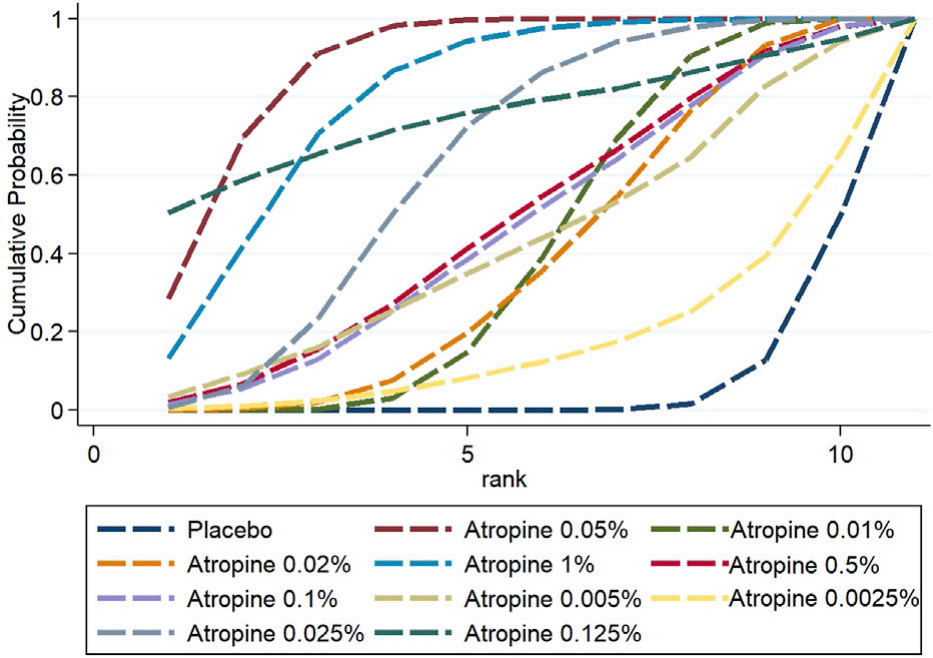
Cumulative probability ranking results for refraction change.

**FIGURE 10:**
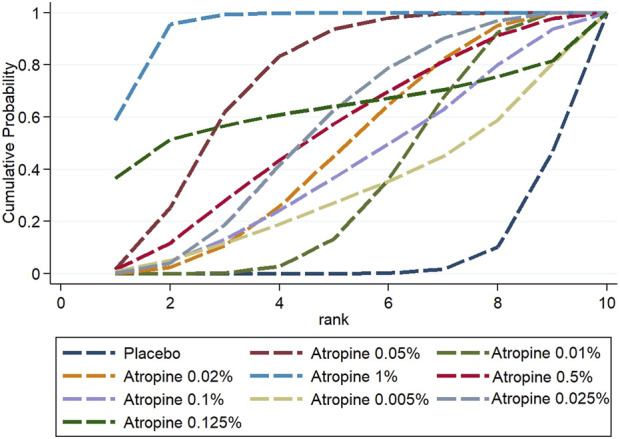
Cumulative probability ranking results for axial length change.

In contrast, when considering changes in axial length, 1% atropine emerged as the most efficacious with a SUCRA value of 95.3%, ranking it first. The ranking for the change was: 1% atropine, followed by 0.05% atropine (73.7%), 0.125% atropine (60.4%), 0.5% atropine (54.7%) which was equally effective as 0.025% atropine (54.7%), 0.02% atropine (47.3%), 0.1% atropine (41.2%), 0.01% atropine (34.8%), 0.005% atropine (31.2%), and finally placebo at 6.9%. These rankings are visually represented in Figures 9, 10.

#### 3.3.5 Cluster analysis

**FIGURE 11:**
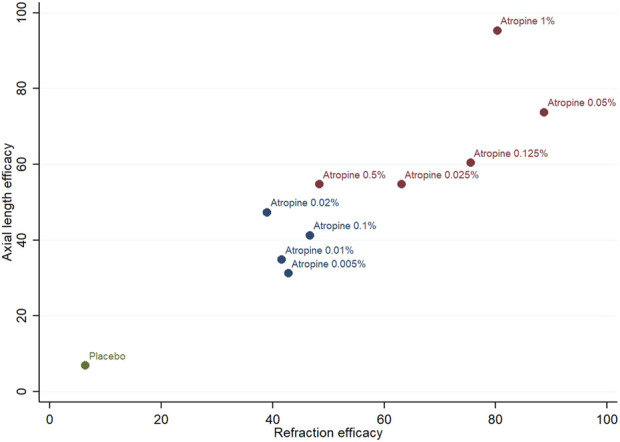
Cluster analysis plot for refraction efficacy and axial length efficacy.

To determine the most effective atropine concentration for controlling myopia progression, cluster analysis was conducted on the key outcome measures: refraction and axial length changes. The analysis revealed that the 1% atropine concentration was the most distant from the origin (zero point) in the two-dimensional coordinate system, indicating its superior efficacy in delaying both refraction and axial length increases. Conversely, the placebo was found to be the closest to the origin, indicating the least effective treatment in this context. These findings are visually depicted in Figure 11.

#### 3.3.6 Publication bias

**FIGURE 12:**
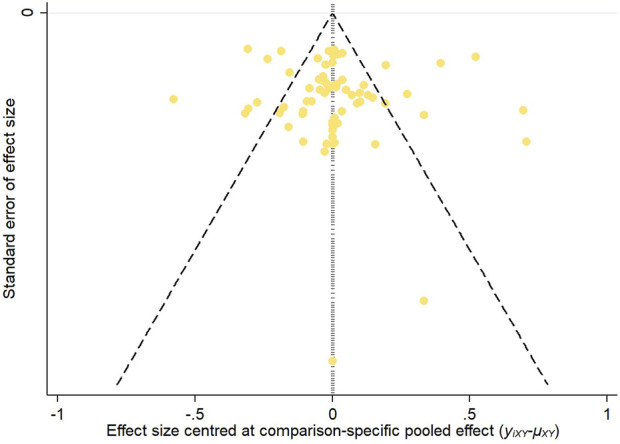
Inverted funnel plot of refraction change.

**FIGURE 13:**
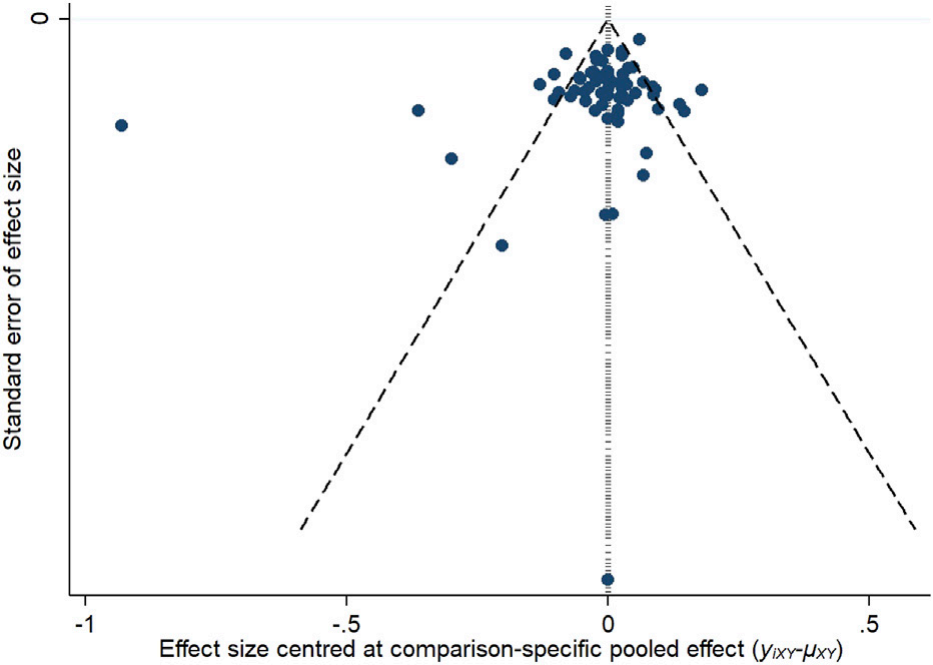
Inverted funnel plot of axial length change.

To assess potential publication bias, funnel plots were constructed based on two key outcome measures: changes in refraction and axial length. The distribution of the studies across the funnel plots indicated a generally symmetrical pattern around the midline, suggesting that the majority of the clinical efficacy studies were consistent in their findings. However, the presence of some studies at the lower end of the funnel, falling outside the 95% confidence interval boundaries, raised concerns about possible publication bias and the influence of small sample sizes. Egger’s test showed that the *p*-value was 0.4763 (>0.05) in refraction change, which indicated that there was no significant publication bias or small-sample effect; and the *p*-value was <0.05 in axial length change, which indicated that there was publication bias. These observations are illustrated in Figures 12, 13.

## 4 Discussion

In this study, a network meta-analysis was conducted on 39 papers to evaluate the effectiveness of 10 different atropine concentrations in delaying myopia progression in East, South and Southeast Asian children. The analysis revealed that five concentrations—0.05%, 0.01%, 0.02%, 1%, and 0.025% atropine—were more effective in delaying refraction change compared to other concentrations. Similarly, these concentrations also showed comparable efficacy in slowing the progression of the axial length.

When ranking the efficacy of these atropine concentrations, 0.05% atropine emerged as the most effective in slowing refraction change, while 1% atropine was found to be the most effective in slowing the progression of axial length. The combined efficacy in terms of both refraction and axial length indicated that 1% atropine was superior, followed by 0.05% and 0.125% atropine.

Despite its high efficacy, 1% atropine is associated with a higher incidence of post-dose adverse effects and a more rapid rebound of myopia after treatment cessation, as noted in previous studies (Kumaran et al., 2015; Wu et al., 2019; Gan et al., 2022). This indicates that 1% atropine may not be the optimal concentration. An adverse effect is a harmful reaction unrelated to the therapeutic purpose that occurs during the prevention, diagnosis or treatment of a disease when the drug is used according to normal usage and dosage. Common adverse effects are seen in the use of atropine solution to delay myopia: photophobia, poor near visual acuity, allergy, chalazion and systemic effects. Post-medication myopia rebound is a phenomenon in which myopia is effectively controlled or slows down during treatment with atropine solution, but after stopping the medication, myopia increases again to a certain extent due to various factors. Several studies have shown that the higher the concentration of atropine, the more likely it is that adverse effects or myopia rebound will occur (Kumaran et al., 2015; Gong et al., 2017). A meta-analysis showed that the incidence of photophobia during the treatment period was 43.1% for atropine at concentrations of 0.5%–1%, compared with only 17.8% for atropine at concentrations of 0.01%–0.5% (Gong et al., 2017).The ideal atropine concentration should balance efficacy and safety, not merely focusing on the former (Ha et al., 2022). 0.05% atropine, which is the second most effective, offers efficacy comparable to 1% atropine but with a lower incidence of adverse effects and a slower myopia rebound rate after discontinuation (Gong et al., 2017; Zhu Q. et al., 2023). This makes 0.05% atropine a potential optimal concentration for myopia control in children in East, South and Southeast Asia.

Furthermore, this study finds that there may be no significant difference between 0.5%, 0.1%, 0.005%, 0.0025% atropine, 0.125% atropine, and placebo in terms of delaying refractive error and axial length progression. This finding contrasts with other studies (Wu et al., 2019; Ha et al., 2022). The inconsistency could be attributed to the limited number of studies that included these atropine concentrations and the potential influence of small sample sizes. Small sample effects and publication bias may lead to overestimate the true effect of a treatment and increase the uncertainty of the results, thereby interfering with the overall findings. Thus, the results from this study should be viewed with caution. Future research should prioritize higher-quality studies to ascertain the true efficacy of these atropine concentrations.

Low-concentration atropine, which includes 0.05% atropine, slows myopic progression primarily by reducing axial length growth, and it does not cause significant changes in other ocular biometric parameters, which reduces the incidence of ocular complications (Li et al., 2020; Yam et al., 2022). Although 0.05% atropine is the optimal concentration for balancing effectiveness and safety, 0.05% atropine can also produce adverse effects such as photophobia. To reduce the incidence and magnitude of adverse effects, UV photochromic lenses can be worn along with the medication (Qin et al., 2023). Regarding when to stop using atropine, some studies suggest that it is better to stop using atropine at an older age, when myopia progression is minimized. Before discontinuing atropine, atropine concentrations should be gradually reduced until the time of discontinuation (Yam et al., 2022).

The exploration of 0.01% atropine as a treatment for controlling myopia in children has gained significant interest. It maintains effectiveness while minimizing side effects and the rebound of myopia after treatment cessation, as well as facilitating a quicker recovery of pupil diameters affected by the medication (Chia et al., 2014; 2023). However, the suitability of 0.01% atropine is not universal among myopic children (Wang et al., 2020). Research indicates that Asian myopic children and those of Asian descent tend to respond better to 0.01% atropine than Caucasian children, possibly due to differences in iris melanin content (Jeon et al., 2022). Additionally, the age of onset of myopia influences the effectiveness of atropine, with earlier onset associated with reduced efficacy. Furthermore, children with high myopia or a family history of high myopia, as well as those with rapid myopia progression, may not respond as well to low concentrations of atropine (Clark and Clark, 2015; Jeon et al., 2022). Interestingly, some studies propose that 0.01% atropine may have a preventive effect on myopia development in non-myopic children who are at high risk. In summary, 0.01% atropine is deemed appropriate for older children of Asian or Asian descent with low initial myopia and for myopia prevention in at-risk children. However, the optimal duration of treatment, continuation, and cessation remain unclear and warrant further investigation.

The progression of myopia in adolescents is influenced by a multitude of factors, including genetic, environmental, gender, and ocular parameters (Yu et al., 2023). Genetic predisposition plays a role, with children from families with a history of myopia being more susceptible to developing myopia (Xiao et al., 2022). In addition to this, genetic differences are also reflected in the efficacy of atropine in the treatment of myopia. Atropine is more effective in controlling the progression of myopia in Asian children compared to white children. The exact mechanism by which atropine slows the progression of myopia is yet unknown, but some research indicates that it primarily affects the retina’s melanocytes, which among other things release new molecules that prevent scleral expansion and slow the progression of myopia (Zhao and Hao, 2021). The effectiveness of atropine may be impacted by the quantity of melanin in the iris, which can differ between races (Fu et al., 2020). Moreover, it may also be related to the fact that Asian children themselves have a high degree of myopia, which progresses more rapidly. Environmental factors, particularly in East, South and Southeast Asia, contribute to a higher prevalence of myopia among children, potentially linked to the economic and educational standards of the region (Chen et al., 2024). Children from low-income countries and those from highly educated environments are more likely to be affected by myopia. This may be related to time spent outdoors and time spent using electronic screens. Prolonged close work and increased screen time, are positively correlated with myopia development, while outdoor activities have a negative correlation (Liu et al., 2024; Zhang X.B. et al., 2024). In addition, female children appear to have a higher incidence of myopia than males (Ying et al., 2024). Ocular characteristics, including smaller refractive error, longer axial length, lower accommodation, thinner lens, greater anterior chamber depth, and larger pupil diameter, are also associated with myopia development. Identifying these risk factors is crucial for targeting high-risk individuals for prevention and early intervention strategies to reduce the prevalence of myopia (Zhu S. et al., 2023).

This study represents one of the first network meta-analyses assessing the efficacy of atropine concentrations for myopia control in this specific demographic. Despite its contributions, the study has several limitations. Firstly, it focused solely on the efficacy of atropine concentrations in managing myopia, neglecting to conduct a network meta-analysis on their safety. This omission was due to the scarcity of studies reporting adverse events and the rebound of myopia following treatment cessation. There may be 2 reasons for this: first, before, most researchers focused mainly on the efficacy of atropine in delaying myopia, and few researchers focused on adverse effects and myopia rebound; moreover, the study period of atropine in delaying myopia is long, usually more than 6 months, and some studies lasted for up to 4 years, and the determination of adverse effects relies on the chief complaints of patients or patients’ families, which may generate recall bias, which increases the difficulty of collecting information. Going forward, there is a need for researchers to focus on the adverse effects and myopic rebound phenomenon produced by atropine, which will help in the comprehensive evaluation of atropine. Researchers can reduce recall bias by increasing the number of observations and shortening the duration of observations to make the data collected more accurate and reliable. Secondly, the analysis of publication bias demonstrated the potential influence of small sample sizes, necessitating a cautious interpretation of some findings. Additionally, the results indicate heterogeneity across studies, it is important to acknowledge that both known and unknown confounding factors, such as the age of initial myopia onset and family history of high myopia, could impact the efficacy of atropine. These factors could ideally be explored through subgroup analysis, but this was not feasible due to inadequate reporting in the existing literature. As different atropine concentrations become more widely available, it is anticipated that future research will provide more comprehensive insights, ultimately leading to a more definitive consensus on the optimal concentration for atropine use.

## 5 Conclusion

This study presents a network meta-analysis of 39 research papers focusing on the role of atropine concentrations in controlling myopia among children in East, South and Southeast Asia. It assessed 10 different concentrations of atropine, ranking their efficacy in terms of refraction and axial length. The analysis indicated that the 1% atropine concentration demonstrated the highest combined efficacy. However, when weighing efficacy against safety, 0.05% atropine emerges as a preferable option for myopia management in this demographic. It is important to recognize that 0.01% atropine, while popular, is not universally suitable for all myopic children. Customized treatment protocols tailored to individual characteristics are essential for optimal outcomes.

Looking ahead, there is a clear need for more extensive, multi-center, large-sample randomized controlled trials. These studies should aim to thoroughly investigate the adverse effects of atropine concentrations and the potential rebound effect following drug cessation. Researchers can reduce recall bias by increasing the number of observations and shortening the duration of observations to make the data collected more accurate and reliable. It is also not known whether there are potential long-term effects of atropine on children’s eye health. Long-term follow-up studies are needed to explore this question. Such research will enable a more comprehensive and nuanced evaluation of atropine concentrations for myopia control.

## Data Availability

The original contributions presented in the study are included in the article/Supplementary Material, further inquiries can be directed to the corresponding authors.
