# Supplementary material for: Effectiveness of various atropine concentrations in myopia control for Asian children: a network meta-analysis
Source: Front Pharmacol. 2024 Dec 16;15:1503536. doi: 10.3389/fphar.2024.1503536 (PMC11683068; doi:10.3389/fphar.2024.1503536)
Supplement: Supplementary file 1 [file DataSheet1.docx]

Supplementary Material

# Supplementary Figures and Tables

## Supplementary Figures


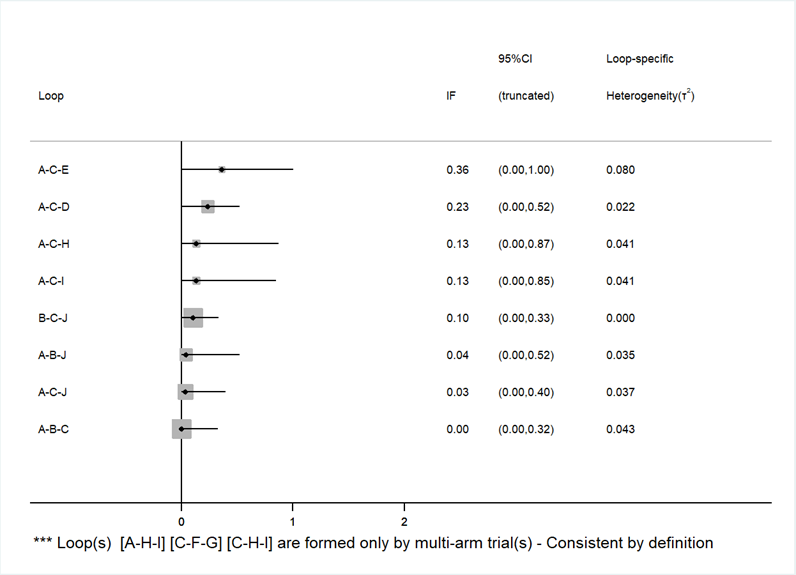


**Supplementary Fig.1** The loop inconsistency test result for refraction change. (Note: A, Placebo; B, Atropine 0.05%; C, Atropine 0.01%; D, Atropine 0.02%; E, Atropine 1%; F, Atropine 0.5%; G, Atropine 0.1%; H, Atropine 0.005%; I, Atropine 0.0025%; J, Atropine 0.025%; K, Atropine 0.125%.)


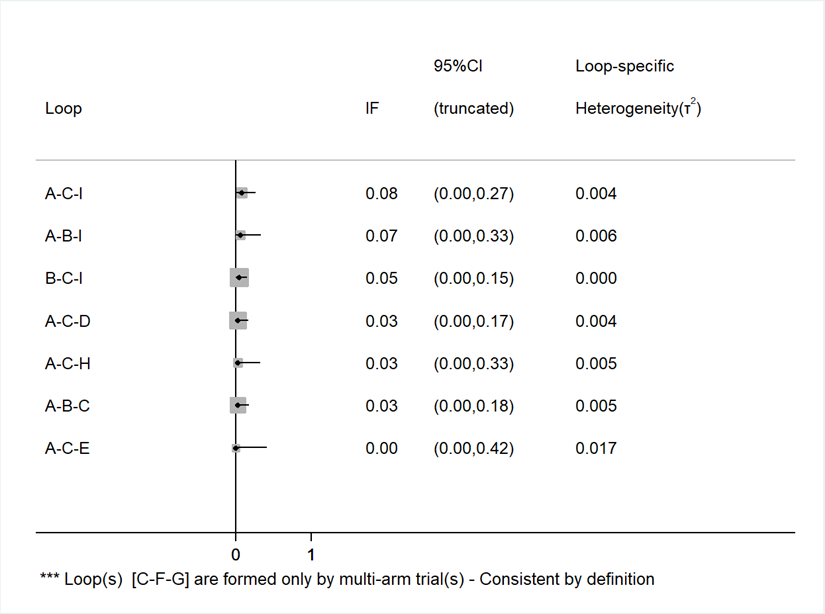


**Supplementary Fig.2** The loop inconsistency test result for axial length change. (Note: A, Placebo; B, Atropine 0.05%; C, Atropine 0.01%; D, Atropine 0.02%; E, Atropine 1%; F, Atropine 0.5%; G, Atropine 0.1%; H, Atropine 0.005%; I, Atropine 0.025%; J, Atropine 0.125%.)

## Supplementary Tables

**Supplementary Table 1** Search strategies and results for PubMed.

| PubMed | | |
| --- | --- | --- |
| Search number | Search formula | Search results |
| #1 | ((((atropine[MeSH Terms]) OR (atropine[Title/Abstract])) OR (atropinol[Title/Abstract])) OR (atropine sulfate[Title/Abstract])) OR (cholinergic antagonists[Title/Abstract]) | 42,076 |
| #2 | ((((myopia[MeSH Terms]) OR (myopia[Title/Abstract])) OR (nearsightedness[MeSH Terms])) OR (nearsightedness[Title/Abstract])) OR (shortsightedness[Title/Abstract]) | 29,829 |
| #3 | #1 AND #2 | 628 |

**Supplementary Table 2** Search strategies and results for Web of Science.

| Web of Science | | |
| --- | --- | --- |
| Search number | Search formula | Search results |
| #1 | (((TS=(atropine)) OR TS=(atropinol)) OR TS=(atropine sulfate)) OR TS=(cholinergic antagonists) | 35,205 |
| #2 | ((TS=(myopia)) OR TS=(nearsightedness)) OR TS=(shortsightedness) | 25,858 |
| #3 | #1 AND #2 | 811 |

**Supplementary Table 3** Search strategies and results for Cochrane Library.

| Cochrane Library | | |
| --- | --- | --- |
| Search number | Search formula | Search results |
| #1 | MeSH descriptor: [Myopia] explode all trees | 1,684 |
| #2 | (myopia):ti,ab,kw OR (nearsightedness):ti,ab,kw OR (shortsightedness):ti,ab,kw | 3,695 |
| #3 | #1 OR #2 | 3,695 |
| #4 | MeSH descriptor: [Atropine] in all MeSH products | 1,368 |
| #5 | (atropine):ti,ab,kw OR ((atropinol):ti,ab,kw OR (atropine sulfate):ti,ab,kw OR (cholinergic antagonists):ti,ab,kw | 5,045 |
| #6 | #4 OR #5 | 5,048 |
| #7 | #3 AND #6 | 355 |

**Supplementary Table 4** Search strategies and results for EMBASE.

| EMBASE | | |
| --- | --- | --- |
| Search number | Search formula | Search results |
| #1 | atropine:ab,ti OR atropinol:ab,ti OR 'atropine sulfate':ab,ti OR 'cholinergic antagonists':ab,ti | 36,416 |
| #2 | myopia:ab,ti OR nearsightedness:ab,ti OR shortsightedness:ab,ti | 26,641 |
| #3 | #1 AND #2 | 706 |
